# Pennsylvania Hospital—Surgical Wards—Service of Dr. Norris

**Published:** 1842-03-19

**Authors:** E. Hartshorne

**Affiliations:** Resident


### *Pennsylvania Hospital—Surgical Wards—Service of Dr. Norris.*

By Dr. E. Hartshorne, Resident

*Luxation and Fracture of the Humerus ; both cured.*—Otto M., aged 7, was admitted January 8th, with luxation anteriorly of the left humerus, complicated with fracture of its surgical neck. This double injury resulted from a blow upon the shoulder, inflicted by a horse in the act of knocking down and running over the child. When presented in the ward, within an hour after the accident, the head of the humerus was found in front just below the outer third of the clavicle and behind the upper margin of the pectoralis major muscle. The shoulder was flattened, and the arm, which dangled powerless by the side, with the lower fragment drawn somewhat inwards, could be moved in every direction without materially affecting the position of the head of the bone. Crepitation between the fragments and a depression on the outside of the limb above the insertion of the deltoid, corresponding to the inward displacement of the lower fragment, together with loss of power in the extremity, and mobility at a point below the natural articulation, amply proved the existence of a fracture complicating the evident luxation already discribed.

Great pain was experienced, but no inflammation had yet arisen in the parts. The dislocation was immediately reduced by extension of the limb downwards and slightly outwards, aided by manipulation of the disarticulated head in such a manner as to thrust it into place. The muscles being relaxed and feeble offered little resistance in this operation. The fragments were then adjusted in apposition, and a well padded jointed splint, fixed at a right angle, was applied to the extremity, from the axilla to the finger ends, the whole being confined to the body in a sling. Thus dressed, the little patient was kept at rest in bed with his arm supported on some cotton and a pillow. During the first two weeks the slightest touch upon the part, on handling of the arm, or even raising the body of the child in bed, appeared to create much suffering, but no violent inflammation appeared to exist at any time. After this period, however, the patient made no more complaint, and union seemed rapidly advancing. The angle of the splint was gradually increased, and daily passive motion of the elbow joint was commenced on the 16th day. Consolidation appeared to be complete within 18 days, and on the 20th the splint was laid aside. Discharged cured February 5th, the use of the shoulder being unimpaired.

